# Prescribing pattern of statins for primary prevention of cardiovascular diseases in patients with type 2 diabetes: insights from Ethiopia

**DOI:** 10.1186/s13104-019-4423-9

**Published:** 2019-07-09

**Authors:** Gebre Teklemariam Demoz, Shishay Wahdey, Gebremicheal Gebreslassie Kasahun, Kalay Hagazy, Daniel Gebrehawaria Kinfe, Hagos Tasew, Degena Bahrey, Yirga Legesse Niriayo

**Affiliations:** 1 School of Pharmacy, College of Health Sciences, Aksum University, PO.Box: 298, Aksum, Ethiopia; 2 School of Public Health, Mekelle University, Mekelle, Ethiopia; 3 School of Medicine, Aksum University, Aksum, Ethiopia; 4 Nursing School, Aksum University, Aksum, Ethiopia; 5 School of Pharmacy, Mekelle University, Mekelle, Ethiopia

**Keywords:** Type 2 diabetes, Statin, Prescribing pattern, Primary prevention, Ethiopia

## Abstract

**Objective:**

Although most clinical practice guidelines endorsed statin use in type 2 diabetes (T2D) patients for reducing cardiovascular diseases (CVD), little is known about statin utilization in case of Ethiopia. Hence, this study was aimed to evaluate prescribing pattern of statins for primary prevention of CVD in T2D patients. A retrospective study conducted in T2D patients with the age group of 40–75 years. Prescriptions were audited for details of statin use and dose intensity. Descriptive analysis was performed using SPSS version 22.0.

**Results:**

We included a total of 323 study subjects. Of those, 55.7% study subjects were found to be received statin for their primary prevention of CVD. Commonly prescribed type of statins was simvastatin (37.2%), atorvastatin (32.8%) and rosuvastatin (15.6%). Low, moderate and high intensive dose of statins were prescribed in 27.8%, 46.1%, and 26.1%, respectively. Of those subjects received statin, 60.6% had on target cholesterol level. Overall, a significant percentage of subjects did not receive their recommended statin for primary prevention of CVD which is below the guidelines’ recommendation. Therefore, adherence to guidelines may help to promote the use of statins for primary prevention of CVD in T2D and advance interventions to improve statin prescribing should be considered.

**Electronic supplementary material:**

The online version of this article (10.1186/s13104-019-4423-9) contains supplementary material, which is available to authorized users.

## Introduction

Cardiovascular diseases (CVD) is the leading cause of morbidity and mortality in patients with T2D [1]. Patients with T2D historically have two or three times higher rate of CVD than adults without diabetes [2]. No doubt the benefits of statins in secondary prevention of CVD have been explicitly established [3–6]; increasing evidence supports the role of statins in primary prevention of CVD as well [7, 8]. Patients with T2D between 40 and 75 years, Low Density Lipid (LDL) of 70–189 mg/dL and without coronary artery disease or stroke are ideal candidates to receive statin therapy as a primary prophylaxis [9]. Furthermore, reducing high blood cholesterol, a risk factor for CVD in people with and without a past history of CVD is an important goal of pharmacotherapy. Statins are the drugs of choice for cholesterol lowering and cardioprotection. Thus, statins use should be considered in all people with T2D over 40 years of age [4, 10].

According to American Diabetes Association (ADA) standards of care recommend moderate-intensity statins for all T2D patients between the age of 40 and 75 years as a primary prevention. This evidence is strong for those patients with the age group of 40–75 years, represented statin use showing benefit [11]. Moreover, the American College of Cardiology/American Heart Association (ACC/AHA) clinical practice guidelines also suggest that patients 40–75 years of age with T2D and an LDL-C level of ≥ 70 mg/dL, moderate-intensity statins is required without calculating 10-year ASCVD risk [12]. Formal risk estimation is unnecessary in people with T2D; since they are all at high risk of CVDs; thus proper uses of statins decrease the risk of coronary heart disease (CHD) in patients with T2D and hyperlipidemia [13].

The benefits of statins use for primary prevention of CVD have been reported in numerous studies [14]. The Collaborative Atorvastatin Diabetes Study (CARDS), [15] reported a significant reduction in CV event rate in this specific population. Indeed, another meta-analysis shows that statins are effective agents that can reduce major coronary events by 21% and stroke by as much as 36% in diabetic patients [16].

Therefore, the rate of statin utilization increased progressively over the last 11-year period to an overall peak of 31.8% [17]. However, statically a low percentage of patients with T2D were prescribed a statin (35.1%) [18]. One study conducted in United States large medical-care reported that 40% with diabetes had filled statin prescription [19]. Another interesting study was conducted in China indicated only 33.8% of patients with T2D received one or more lipid-lowering agents for the primary prevention of cardiovascular events [7]. In India statins were prescribed in 55.2% of patients with T2D [20]. In addition in Malaysia, 65% had a statin therapy prescription for primary prevention in T2D [21].

Although utmost contemporary clinical practice guidelines endorsed that statin use in those T2D patients with the age group between 40 and 75 years for reducing cardiovascular risks and all-cause mortality are proven significantly, underutilization of statins is reported in the sub-Saharan African countries [22, 23]. In Ethiopia, the escalating burden of CVD and its risk factors warrants for timely action and could be scaled up at a modest budget increase [24]. However, little is known about statin utilization in case of Ethiopia for primary prevention of CVD. Therefore, the primary purpose this study was aimed to evaluate the prescribing pattern of statins for primary prevention of CVD in T2D patients. This study may add to the previous scientific works by providing present-day data on the prescribing rates of statins for patients with T2D in primary prevention of CVD.

## Main text

### Methods

#### Study subjects and data collection procedures

A 3 years retrospective study was conducted from July 2015 to June 2018. About 2360 outpatients’ who were objectively diagnosed with diabetes chart were reviewed. Out of these, 323 subjects were included in the final analysis (Additional file 1). The study subjects were selected using systematic random sampling technique. The inclusion criteria were confirmed cases of T2D aged between 40 and 75 years, outpatients with T2D who had regular follow-up care of the clinic. Patients who had known history of myocardial infarction, acute coronary syndrome or stroke, angina, and patients with any other CHD were excluded from our study.

Data were collected from patients’ medical chart which were included questions about sociodemographic and clinical details (includes diabetes complications, co morbidities, duration of diabetes, laboratory values and medication profiles). Here we evaluate whether statins should be used routinely for primary prevention of CVD in patients with T2D between the age of 40 and 75 irrespective of other risk factors.

Prescriptions were audited for different medications including statins, antidiabetic, and antihypertensive drugs. All types of prescribed statins were evaluated, while other forms of lipid-lowering agents (e.g., fibrates) were not included due to unavailability. We focused on the details of type of statin, up titration/equivalence dose and daily dose in mg/day. According to the 2017 ACC/AHA guideline [12], statins were grouped into three levels of dose intensity based on their ability to lower LDL (low-intensity, moderate-intensity and high-intensity statins). (1) low intensity statins: atorvastatin < 10 mg/day, rosuvastatin < 5 mg/day, simvastatin < 20 mg/day, and lovastatin < 40 mg/day (2) moderate-intensity statins: 10 mg/day ≤ atorvastatin < 40 mg/day, 5 mg/day ≤ rosuvastatin < 20 mg/day, 20 mg/day ≤ simvastatin < 80 mg/day, lovastatin ≥ 40 mg/day and (3) high-intensity statins: atorvastatin ≥ 40 mg/day, rosuvastatin ≥ 20 mg/day and simvastatin ≥ 80 mg/day.

#### Data analysis

Data were entered and analyzed using EpiData Manager Version 4.0.2.00 (EpiData Association, Denmark) [25] and SPSS version 22.0 (SSPS Inc., Chicago, Illinois, USA), respectively. Demographics, clinical characteristics, and statin utilization were analysed and present categorical variables as percentages and continuous variables as means (standard deviations) or their 95% confidence intervals as appropriate.

### Results

#### Socio-demographic and clinical characteristics

A total of 323 study subjects were included in this study. Nearly half (51.4%) of the study subjects were females. More than half (58.2) of the study subjects were between 40 and 65 years of age. The mean diabetes duration since diagnosis of subjects were (11.9 ± 6.9) years. Detail baseline socio-demographic and clinical characteristics including laboratory parameters of the study subjects are summarized in Table 1.

**Table 1:** Baseline demographic and clinical characteristics of patients with T2D in Ethiopia, 2018

Category	Subcategory	Statin use (N = 323)		Total (%)	*P* value
		Not received	Received		
Sex	Male	64 (44.8)	93 (51.7)	157 (48.6)	0.57
	Female	79 (55.2)	87 (48.3)	166 (51.4)	
Age group	40–64	86 (60.1)	102 (56.7)	188 (58.2)	0.33
	65–75	57 (39.9)	78 (43.3)	135 (41.8)	
BMI (kg/m^2^)	Mean (± SD)	26 ± 3	26 ± 3	28.23 ± 2.3	0.161
Duration of diabetes	Mean (± SD)	11.03 ± 6	13.12 ± 7	11.9 ± 6.9	0.051
Presence of comorbidities	Yes	77 (53.4)	175 (97.2)	250 (77.9)	0.115
	No	66 (46.2)	5 (2.8)	71 (22.1)	
Types of co morbidities	Hypertension	60 (72.3)	112 (63.6)	172 (66.4)	0.169
	Dyslipidemia	7 (9)	148 (84.6)	155 (61.3)	0.000
	IHD	3 (3.8)	30 (17.0)	33 (13.0)	0.004
	Others^a^	13 (16.5)	32 (18.3)	45 (17.7)	0.724
Presence of complications	Yes	56 (35.5)	58 (32.2)	108 (33.6)	0.061
	No	91 (64.5)	122 (67.8)	213 (66.4)	
Types of complications	Neuropathy	42 (75.0)	40 (61.5)	82 (67.8)	0.114
	Nephropathy	7 (12.3)	9 (13.6)	16 (13)	0.824
	Retinopathy	8 (14)	17 (25.4)	25 (20.2)	0.117
FBG, mg/dL	Mean (± SD)	171.9 ± 49.7	178.1 ± 48.1	174.10 ± 48.9	< 0.001
> 130 mg/dL		41 (28.6)	59 (32.8)	241 (74.6%)	0.003
LDL, mg/dL	Mean (± SD)	109.1 ± 33	119 ± 50	115.7 ± 35.6	0.081
≥ 100		181 (74.2)	41 (36.3)	222 (62.2)	0.058
HDL, mg/dL	Mean (± SD)	46 ± 16	44 ± 16	41.8 ± 10.2	0.624
≤ 40, male, ≤ 50, female		121 (49.6)	52 (46.0)	173 (48.5)	0.712
Triglycerides, mg/dL	Mean (± SD)	159 ± 62	94 ± 118	158.2 ± 121.2	0.154
≥ 150		113 (46.3)	33 (29.2)	146 (40.9)	0.081
Total cholesterol, mg/dL	Mean (± SD)	181 ± 34	193 ± 54	165.54 ± 38.3	0.067
≥ 200 mg/dL		68 (27.9)	29 (25.7)	97 (27.2)	0.121
Overall cholesterol level	On target	21 (14.7)	109 (60.6)	130 (40.2)	
	Not on target	122 (85.3)	71 (39.4)	193 (59.8)	0.171
eGFR, mL/min/1.73 m^2^	≤45	31 (12.7)	13 (11.50)	44 (12.3)	0.221
BP (mmHg), mean (± SD)	Systolic BP	144.18 ± 47.24	139 ± 20	151.17 ± 62.07	0.415
	Diastolic BP	84 ± 14	81 ± 9	85.11 ± 0.08	0.281
Systolic/diastolic	>140/90	98 (40.2)	19 (16.8)	117 (32.8)	0.078
Risk factors	Low (T2D alone)	141 (98.6)	93 (51.7)	234 (72.5)	0.031
	Medium (2–3)	2 (1.4)	61 (33.9)	63 (19.5)	0.009
	High (≥4)	0 (0.0)	26 (14.4)	26 (8.0)	0.002

*BMI* body mass index, *FBG* fasting blood glucose, *LDL* low-density lipoprotein, *HDL* high-density lipoprotein, *eGFR* estimated Glomerular Filtration Rate, *BP* blood pressure, *SD* standard deviation

^a^Thyroid disorders, peptic ulcer disease, asthmatic

Hypertension was present in 66.4%, with a blood pressure (BP) > 140/90 mmHg in 32.8% subjects. A total cholesterol ≥ 200 mg/dL in 27.2%, LDL ≥ 100 mg/dL in 62.2%, triglycerides ≥ 150 mg/dL in 40.9%, and HDL in 48.5%. Of those subjects who received statin therapy, 60.6% of subjects were found to be on target cholesterol level. Moreover, diabetic complications such as neuropathy, retinopathy and nephropathy with eGFR ≤ 45 mL/min/1.73 m^2^ in 12.3%, was seen in 67.8%, 20.2%, and 13.0%, respectively.

#### Prescribed statins and other medication profiles

**Fig. 1:**
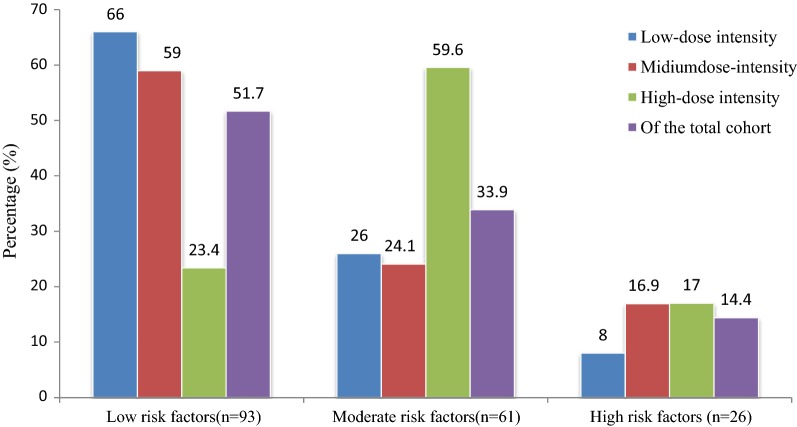
Risk factors in relation to statin dose–intensity among patients with T2D, 2018

Prescribed statins and others medications are shown in Table 2. Statins were prescribed in 180 (55.7%) study subjects. The most frequently prescribed type of statin prescriptions was simvastatin (37.2%), followed by atorvastatin (32.8%) and rosuvastatin (15.6%). Of those subjects received statins (n = 180), low-dose statins were prescribed in 27.8%, moderate dose in 46.1%, and high dose in 27.8%. Likewise, high-dose statins were prescribed in the high risk (17.0%), medium-risk (59.6%), and low-risk (23.4%) groups (Fig. 1). Furthermore, oral glucose lowering drugs alone and insulin alone was prescribed in 53.9% and 17.8%, respectively.

**Table 2:** Prescribing pattern of statins and other medications among patients with T2D in Ethiopia, 2018

Variables	Subcategories	Frequency	Percent
Antidiabetic agents	OGLD alone	173	53.9
	Insulin alone	57	17.8
	OGLD + insulin	91	28.3
Antihypertensive agents	ACE inhibitors	157	47.4
	Beta-blockers	128	39.6
	Calcium channel blockers	39	12.1
	Diuretics	69	21.5
Antiplatelets	Aspirin	35	10.8
	Clopidogrel	5	1.5
Lipid lowering agents	Statins	180	55.7
Prescribed type of statins	Simvastatin	67	37.2
	Atorvastatin	59	32.8
	Rosuvastatin	28	15.6
	Lovastatin	26	14.4
Statins in various dose-intensity	Low intensity	50	27.8
	Moderate intensity	83	46.1
	High intensity	47	26.1
Source of medication (n, %)	For free	188	58.2

*ACE* angiotensin converting enzyme, *T2D* type 2 diabetes

### Discussion

The current study sought to provide an up-to-date data on statin prescription utilization for the primary prevention of CVD in subjects with T2D who had no history of CVD in Ethiopia. We evaluated the prescription pattern for specific statin regimens in patients with T2D for primary prevention of CVD. In the present study, the prescription audit shows that statins are prescribed in 55.7% (95% CI 50.2–61.9) of patients with T2D. Simvastatin (37.2%), atorvastatin (32.8%) and rosuvastatin (15.6%) are commonly prescribed types of statins. Moderate-dose of statins are prescribed in less than half (46.1%) of the study subjects.

The present result was consistent with the study reported from India, 55.2% [20]. Whereas compared with findings reported from Malaysia, 65% [21] for primary prevention of CVDs in hospitalized patients with T2D, our data show that lower rates of prescription use of statins. In addition, the prescribing pattern of statins for the primary prevention of CVD in patients with T2D of this study was higher than studies from Denmark, 47% [26], USA, 40%, [19] and China, 33.8% [7]. This discrepancy could be explained by the difference in number of risk factors in subjects involved in the study, study design and setting.

In our study more than half (58.2%) of subjects in our study were obtained for free who had authorized by the responsible body. However, because of unavailability patients may went without the prescribed statin and stay until the next visit. In fact, in Africa and many of other middle income nations, patients with diabetes are suffering from unavailability and unaffordability of their drugs resulting to remain the leading barrier in diabetes care [27, 28]. Therefore, this pronounced incidence of suboptimal statin utilization in our study may highlight a need for the prescribers and responsible body to pay more emphasize in prescribing statins to patients with T2D.

Although the prescriptions of statins are significantly greater in high-risk study subjects, the overall prescriptions of statins as well as high-dose statins are suboptimal and much lower than the standard guidelines’ recommendations [9, 12]. This is in contrary to the explicit benefit of statins use for the primary prevention of CVD in patients with T2D that contemporary standards of care guidelines recommend, moderate-dose intensity of statins for all T2DM patients between the age of 40 and 75 years [9, 11, 12].

The implication of statins prescription use also supported by a large randomized controlled trial aimed to show the usefulness of statin for the primary prevention of CVD in subjects with T2D who had at least one or more of CVD risk factors, also showed a superior risk reduction of CVD events in 37% of statin users [14]. Thus, it seems to suggest that some level of clinical inertia, where prescribers might be slow in responding to the clinical parameters. The prescribing practice of statins showed that there is a need to intensify statin use in compliance to the contemporary clinical guidelines’ recommendations.

Furthermore, the present study indicates simvastatin was the most commonly prescribed type of statin, followed by atorvastatin and rosuvastatin. In contrary, finding reported from India [20] shows atorvastatin was the most commonly prescribed type of statin (74.1%), followed by rosuvastatin (29.2%). Unfortunately, moderate-dose of statins is recommended for all patients with T2D with the age of 40–75 years [9, 11, 12]. In our study we found that moderate dose intensity are prescribed in less than half (46.1%) of the study subjects. Likewise, majority (65%) of prescribed statins were maintained on the same dose with a very small number (8%) had their dosages titrated upwards. In addition, only 6% of study subjects had switched with appropriate dose of equivalency.

Interestingly, the present study found that of those subjects who received statin therapy, only 60.6% of subjects were found to be on target cholesterol level. Certainly, only nearly two-fifth (39.5%) of study subjects of this study had detailed lipid profile. This indicates noncompliance with the standard guidelines about monitoring of lipid profile that all patients with T2D should be tested at least annually [9]. This might also be the reason for: of those subjects who received statins, only 60.6% had on target cholesterol. Thus, need to emphasize periodic monitoring of their lipid in optimizing the utilization of statins in response to the CVD risks factors, indicating that statin prescription decisions are, at least in part, based on the risk assessment and lipid profile.

Another interesting finding of the present study, compared to subjects who did not receive statin, poor glycemic control had worsened in subjects who received statins (32.8% versus 28.6%). This could be explained due to the fact that simvastatin was the most commonly prescribed type of statin (37.2%) in which simvastatin has the potential effect in reducing insulin secretion and sensitivity [29].

### Conclusions

In conclusion, this study shows that prescriptions of statins in patients with T2D in Ethiopia are suboptimal indicating that utilization of statins among patients with T2D was substantially far below the current clinical guidelines’ recommendation. Efforts in adherence of the contemporary clinical guidelines may help to promote the use of statin to all patients with T2D for primary prevention of CVD is urgently required.

## Limitations of the study

This study was conducted at a single center that may have limitation in generalizability.

The inherent problems associated with being used retrospective study design may also another limitation that statin prescription utilization might depend on other factors not documented in the current registry. Thus, the retrospective nature of the study may limit the generalizability of the evidence that was generated from the study.

## Additional file

**Additional file 1: Figure S1.** Schematic flowchart of participant recruitment for analysis.

## Data Availability

Data that aid the findings of the current study are available from the corresponding author.

## Abbreviations

ADA: American Diabetes Association
CVD: cardiovascular disease
eGFR: estimated Glomerular Filtration Rate
FBG: fasting blood glucose
IDF: International Diabetes Federation
LLAs: lipid-lowering agents
OGLD: oral glucose-lowering drugs
SMBG: self-monitoring of blood glucose
WHO: World Health Organization
