# Supplementary material for: Prescribing pattern of statins for primary prevention of cardiovascular diseases in patients with type 2 diabetes: insights from Ethiopia
Source: BMC Res Notes. 2019 Jul 9;12:386. doi: 10.1186/s13104-019-4423-9 (PMC6617647; doi:10.1186/s13104-019-4423-9)
Supplement: Supplementary file 1 — Additional file 1: Figure S1. Schematic flowchart of participant recruitment for analysis. [file 13104_2019_4423_MOESM1_ESM.docx]

Schematic flowchart of participant recruitment for analysis, 2018

n=326

Excluded

T1DM (n=203)

Age <40 & >75(n=83)

n=610

Excluded

History of CVD (on secondary prevention, n=1750)

People with diabetes at diabetic clinic during study period (n ≈ 2360)

Chart not available (n = 3)

Excluded

Involved in the final analysis (n= **323**)

Figure S1 Schematic flowchart of participant recruitment for analysis

Figure S1 Schematic flowchart of participant recruitment for analysis
